# Adoption of Telemedicine for Dementia Care in Nigeria: Scoping Review

**DOI:** 10.2196/75168

**Published:** 2025-10-27

**Authors:** Abiodun Adedeji, Huseyin Dogan, Festus Adedoyin, Michelle Heward

**Affiliations:** 1 Department of Computing and Informatics, Faculty of Science and Technology, Bournemouth University, Talbot Campus, Fern Barrow, Poole, Dorset, Bournemouth, BH12 5BB, United Kingdom, 44 7424 832233; 2 Department of Psychology, Faculty of Science and Technology, Bournemouth University, Bournemouth, United Kingdom

**Keywords:** telemedicine, dementia care, Nigeria, mHealth, video consultations, remote monitoring, caregiver support, health care accessibility, national telemedicine strategy, mobile health

## Abstract

**Background:**

Dementia is a global health challenge, particularly in Nigeria, where limited health care infrastructure, cultural stigmas, and poor awareness hinder its care. Telemedicine can improve patient outcomes, increase health care access, and support caregivers. However, challenges such as poor internet connectivity, digital literacy, and a lack of integrated strategies hinder its adoption, particularly in rural areas.

**Objective:**

This scoping review aims to evaluate the adoption of telemedicine for dementia care in Nigeria by highlighting existing interventions, their effectiveness, implementation challenges, and contextual barriers. It also draws on global evidence to propose culturally relevant, sustainable strategies.

**Methods:**

A scoping review was conducted using the PRISMA-ScR (Preferred Reporting Items for Systematic Reviews and Meta-Analyses Extension for Scoping Reviews) framework. Peer-reviewed articles were included if they focused on telemedicine or digital health interventions for dementia care in Nigeria or sub-Saharan Africa and published between January 2010 and February 2024. Databases searched included PubMed, Scopus, CINAHL, PsycINFO, Cochrane Library, and Google Scholar. A total of 23 articles met the inclusion criteria.

**Results:**

Among the 23 studies, 10 (43.5%) focused on mobile health apps, 8 (34.8%) on video consultations, and 5 (21.7%) on remote monitoring tools. These interventions improved caregiver support, medication adherence, and access to specialist care. Key barriers included limited digital literacy, poor internet access, and a lack of cohesive national telemedicine policy.

**Conclusions:**

There is an urgent need for an inclusive national telemedicine policy in Nigeria. Interventions such as mobile health, video consultations, and remote monitoring tools show potential to enhance dementia care, reduce caregiver burden, and improve health outcomes.

## Introduction

### Overview

Dementia is a progressive neurodegenerative condition marked by declining memory, cognitive abilities, and daily functioning, and it presents a significant global health burden [1]. According to the World Health Organization, more than 55 million people are currently affected worldwide, with nearly 10 million new cases emerging each year. This number is expected to rise to 74.7 million by 2030 and 131.5 million by 2050, underscoring the urgent need for scalable and sustainable models of care. Although the social and health care implications of dementia are increasingly recognized globally, its impact in culturally diverse and resource-limited settings, such as Nigeria, remains insufficiently examined.

**Figure 1.**
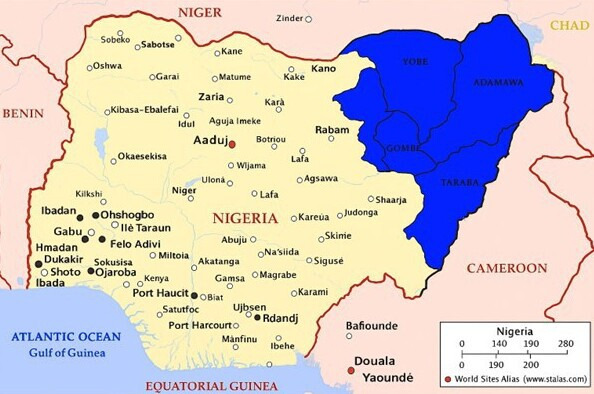
Geographical map highlighting the northeastern Nigerian region for telemedicine dementia care.

Nigeria, Africa’s most populous country, is home to more than 250 ethnic groups and more than 500 languages, yet its adult literacy rate is only 62% with significant urban-rural disparities [2]. The education system faces diglossia, as English contrasts with indigenous languages, affecting health care communication and dementia care. Early-onset dementia is rare but tends to progress more rapidly [2]. Clinical presentations of dementia in Nigeria differ from those in developed countries due to cultural interpretations of aging conditions [3]. Behavioral and psychological symptoms are poorly recognized and underreported due to stigma and lack of awareness in rural areas (Figure 1).

Digital literacy worsens these issues: mobile phone adoption has increased in Nigeria, with more than 70% of the population owning at least 1 device [4]. This highlights the need to address awareness gaps for better telemedicine use in dementia care.

The article evaluates Nigeria’s telemedicine adoption for dementia care, examining sociocultural, linguistic, and technological contexts, while identifying gaps to improve outcomes [5].

### The Role of Telemedicine in Dementia Care

Telemedicine enhances dementia care in Nigeria by providing remote clinical services. According to Louis et al [6], this system includes diagnosis and consultation via mobile phones and computers [7].

Telemedicine allows patients and caregivers to connect with health care professionals, reducing the psychological impacts of distancing [8]. During the pandemic, remote consultations, monitoring, and cognitive assessments eased isolation burdens while strengthening relationships with families and health care professionals, improving well-being and identity [9,10].

Telemedicine use in Nigeria increased during COVID-19, highlighting the need for remote care as face-to-face became limited [11]. Restrictions worsened social isolation and reduced health care access for individuals living with dementia and caregivers [12]. Telemedicine supports diagnosis, caregiver assistance, and community education [10]. In Nigeria, telemedicine provides a viable means to improve dementia care through remote services using computers and mobile phones [13,14].

Dementia remains a global health concern [15]. Increased awareness has emphasized caregiver support, with telemedicine emerging as a key intervention for COVID-19 [15,16].

This review discusses the effectiveness and challenges of telemedicine interventions for dementia care in Nigeria. It also aims to guide future development in resource-poor settings by assessing current usage [17]. Active interventions such as videoconferencing, remote assessment, and mobile apps are prioritized over passive monitoring technologies. According to Haimi [18], research highlights issues such as poor internet connectivity and technological inequality. Despite these issues, telemedicine shows promising potential for improving dementia care in Nigeria and justifies further studies [19].

### Conceptual Framework

The conceptual framework shows interactions between caregivers, health care professionals, individuals living with dementia, and technologies to enhance care delivery [13]. Mobile health (mHealth) app video consultations and monitoring devices improve access and integrate stakeholders into a unified system [20]. Caregivers use telemedicine tools for care management, while health care providers use them for diagnosis, treatment, and follow-up. These tools help overcome geographical barriers [21]. Technologies support caregivers in planning routines and seeking advice [22]. Video consultations address and improve specialist access, and monitoring systems track health for timely intervention [23]. This framework improves health care delivery, reduces caregiver burden, and provides an improved quality of life [24], helping stakeholders identify areas needing improvement.

### Digital Literacy

Digital literacy is essential for telemedicine adoption, especially in resource-poor countries such as Nigeria [25]. It remains a challenge in urban and rural areas [26]. Mobile phone penetration is 70%, but rural digital literacy is about 30% compared to 62% in urban areas, highlighting a digital gap affecting telemedicine [23]. Urban caregivers benefit from better infrastructure, while rural caregivers face limited access, education, and training [27]. Low literacy in rural areas hinders the use of telemedicine tools, such as mHealth or video consultations for specialist care [28,29].

### Challenges of Telemedicine Adoption in Nigeria

Despite the potential benefits, barriers hinder telemedicine use in Nigeria, especially in rural areas [30]. Technological challenges include infrastructure, internet services, and digital literacy, while nontechnological ones involve ethical concerns and funding issues [31]. This scoping review evaluates the adoption of telemedicine for dementia care in Nigeria, highlighting interventions, effectiveness, challenges, and contextual barriers. It draws on global evidence to propose culturally relevant and sustainable strategies for implementation.

## Methods

### Study Design

This study used a scoping review methodology, guided by the PRISMA-ScR (Preferred Reporting Items for Systematic reviews and Meta-Analyses extension for Scoping Reviews) framework. The purpose of this work was to explore the adoption of telemedicine for dementia care in Nigeria by identifying current interventions, implementation challenges, and research gaps.

### Eligibility Criteria

Peer-reviewed articles were eligible for inclusion if they (1) focused on dementia care in Nigeria or sub-Saharan Africa; (2) included individuals living with dementia, caregivers, or health care professionals; (3) reported on telemedicine or digital health interventions (eg, mHealth apps, video consultations, or remote monitoring); (4) were published in English between January 2010 and March 2024; and (5) applied empirical methods, including qualitative, quantitative, or mixed-methods design. Conversely, studies were excluded if they (1) focused on diseases other than dementia, (2) lacked telemedicine components, (3) were purely theoretical or not empirical, or (4) were not published in English (Table 1).

**Table 1.** Inclusion criteria and exclusion criteria for the scoping review.

Criteria	Inclusion	Exclusion
Population	Individual living with dementia, caregiver, or health care professionals	Other diseases than dementia
Interventions	Studies that involve telemedicine interventions or telehealth	Studies that do not involve telemedicine interventions
Study design	Studies that include qualitative, quantitative, and mixed designs	Studies that are purely theoretical
Outcomes measure	The effectiveness of telemedicine on dementia care	No outcomes related to the impact of telemedicine on dementia care
Publication date	Studies published from 2010 to 2024	Studies published before 2010
Language	Studies published in English	Studies published in languages other than English

### Information Sources

The following databases were searched: PubMed, Scopus, CINAHL, PsycINFO, Cochrane Library, and Google Scholar. In addition, citation searching involved backward citation tracking from the reference lists of included articles and relevant systematic reviews to identify further relevant studies.

### Search Strategy

A comprehensive search strategy was developed using keywords such as “telemedicine,” “dementia care,” “digital health,” “remote care,” “caregiver support,” “Nigeria,” and “Sub-Saharan Africa.” Boolean operators and truncations were used to optimize search results. The full search strategy is available in Multimedia Appendix 1.

### Study Selection

Study selection followed a 2-stage screening process in accordance with the PRISMA-ScR framework. In the first stage, 640 records were identified through searches of PubMed, Scopus, CINAHL, PsycINFO, Cochrane Library, and Google Scholar. After removing 220 duplicate records, 420 titles and abstracts were screened independently by 2 reviewers (AA and EO). In the second stage, 100 full-text articles were also independently assessed for eligibility by the same 2 reviewers. At both stages (title or abstract and full-text screening), disagreements were resolved through discussion with a third reviewer (HD).

### Data Charting Process

Data were independently extracted by 2 reviewers (AA and EO) using a standardized data charting form, which was piloted and refined prior to full data extraction. Discrepancies were resolved through discussion, and when necessary, by involving a third reviewer (HD).

### Data Items

The following items were extracted from each included study: authorship, publication year, country, study design, target population, type of telemedicine intervention, measured outcomes, and main findings.

### Synthesis of Results

A narrative synthesis was used to categorize the data into 3 main themes: (1) types of telemedicine interventions, (2) barriers to adoption, and (3) facilitating factors. Where applicable, frequencies and percentages were used to summarize the distribution of study characteristics and intervention types.

### Data Synthesis by Intervention Type

The extracted data were grouped by type of telemedicine intervention. These included mHealth apps, video consultations, and remote monitoring tools. A narrative synthesis was conducted to identify patterns across studies and categorize findings into key intervention types. The goal was to compare their reported outcomes, implementation contexts, and barriers in dementia care delivery.

## Results

### Overview

The reviewed studies explored a variety of telemedicine interventions applied to dementia care within Nigerian or sub-Saharan African contexts. These interventions included mHealth apps, video consultations, and remote patient monitoring systems aimed at supporting caregivers, improving access to specialist care, and enhancing patient outcomes. The included studies used diverse methodological approaches, including qualitative interviews, cross-sectional surveys, randomized controlled trials (RCTs), and mixed methods designs. Terms commonly used across the studies included “telemedicine,” “digital health,” “remote care,” “mHealth,” and “eHealth,” reflecting a broad conceptual framing of digital health delivery in dementia care.

**Figure 2.**
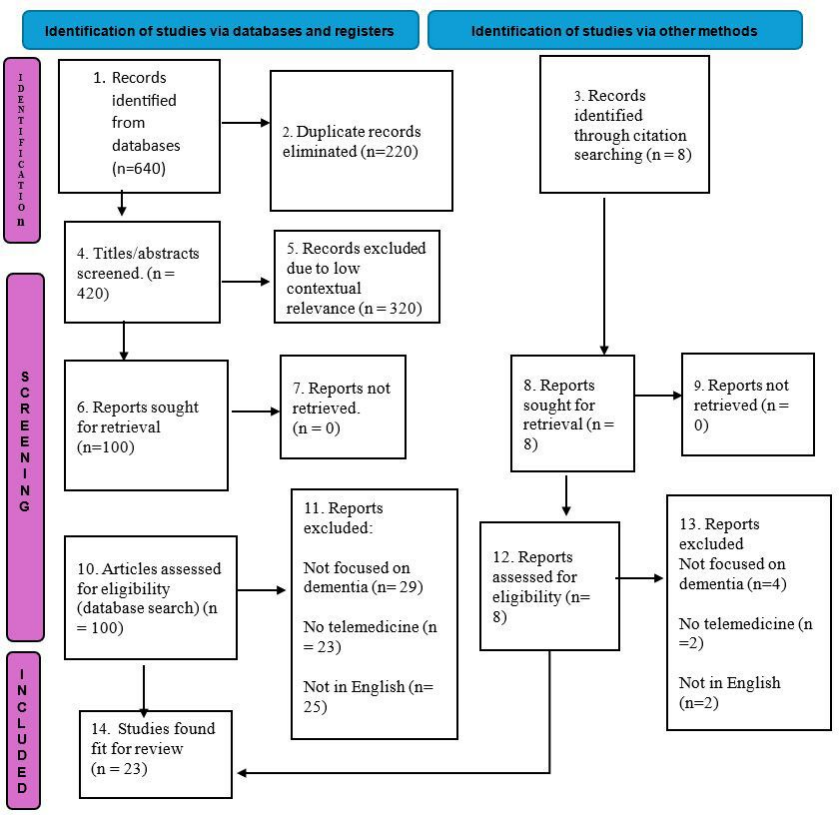
PRISMA-ScR (Preferred Reporting Items for Systematic Reviews and Meta-Analyses Extension for Scoping Reviews) flow diagram showing the selection process.

The study selection process is outlined in Figure 2 below. Out of 640 initial records, 420 were screened, 40 full-text articles were assessed, and a total of 23 studies met the inclusion criteria and were included in the final review. An additional 8 records were identified through backward citation searching of included studies and relevant systematic reviews. All 8 records were retrieved and assessed for eligibility. However, none met the inclusion criteria due to reasons such as not focusing on dementia (n=4), lack of telemedicine components (n=2), and not being published in English (n=2).

### Characteristics of Included Studies

In various regions, the primary characteristics of the studies are outlined in Table 2. A total of 23 studies were conducted mainly in Nigeria (n=7) [1,6,9,11,14,32,33], followed by the United States (n=5) [34-38], the United Kingdom (n=6) [39-44], Hong Kong (n=2) [45,46], Greece (n=1) [13], Germany (n=1) [47], and Spain (n=1) [48]. Study settings varied, individuals’ homes (n=6), health care institutions (n=10), and mixed environments (n=7).

**Table 2.** Characteristics of included studies on telemedicine and dementia care in Nigeria and other sub-Saharan countries.

S.No	Author (year)	Country published	Study design	Intervention type	Sample size (n)	Technology type	Key measure	Outcomes	Modality
1	Muili et al [1] (2023)	Nigeria	Review	Remote consultation, video teleconferencing	—^a^	Cognitive tests, MMSE^b^, HVLT-R^c^, and letter fluency	Feasibility of telemedicine for dementia diagnosis and follow-up	Internet-based	Synchronous
2	Oyinlola et al [11] (2024)	Nigeria	Mixed method	Remote monitoring	—	Barriers and facilitators of telemedicine	Need for national strategy	Internet-based	Asynchronous
3	Louis et al [6] (2021)	Nigeria	Quantitative	Remote monitoring, mHealth^d^	—	Caregiver satisfaction and system usability	Improved usability lowers stress	Smartphone-based	Asynchronous
4	Ibrahim et al [9] (2024)	Nigeria	Mixed method	Video consultations	—	Pandemic-related care disruption	Persistent access during COVID-19 reduced isolation	Internet-based	Synchronous
5	Angelopoulou et al [13] (2022)	Greece	Narrative review	Remote monitoring	—	Feasibility and care efficiency	Improved quality of care and reduced travel burden	Internet-based	Asynchronous & synchronous
6	Anthony [14] (2021)	Nigeria	Case report	Telehealth for rural dementia care	—	Infrastructure barriers	Need for policy framework integration	Internet-based	Synchronous & asynchronous
7	Strini et al [45] (2023)	Hong Kong	Mixed method	mHealth, video telehealth	70	Caregiver burden QoL^e^ metrics	Reduced stress and higher QoL for caregivers	Smartphone-based	Synchronous
8	Page et al [46] (2021)	Hong Kong	Quantitative	Video conferencing for dementia care	60	Cognitive functioning QoL-AD^f^ and caregiver burden	Improved resilience and better scores reduced burden	Internet-based	Synchronous
9	Ojeahere et al [34] (2020)	US	Quantitative	Wearable sensors for dementia monitoring	50	Daily activity tracking for early detection	Improved monitoring of timely interventions	Wearable sensor	Asynchronous
10	Cote et al [39] (2020)	UK	Qualitative	Remote monitoring tools	—	Daily activity tracking and early detection	Improved monitoring of timely interventions	Wearable sensor	Asynchronous
11	Gaugler et al [35] (2021)	US	Systematic review	Care planning, video consultations	250	Cognitive status and care planning	Improved dementia care planning and QoL	Internet-based	Synchronous
12	Yi et al [36] (2021)	US	Qualitative	mHealth for symptom tracking	40	Behavioral symptoms and medication adherence	Improved monitoring reduced hospital visits	Smartphone-based	Asynchronous & Synchronous
13	Mason et al [37] (2022)	US	Cross-sectional	Video telehealth for veterans	24	Semi-structured interviews	Reduced travel burden for caregivers	Internet-based	Synchronous
14	Gately et al [32] (2019)	Nigeria	Qualitative	Telemedicine in hospitals	7	Implementation issues	Improved medication adherence and caregiver satisfaction	Internet-based	Synchronous
15	Adenuga et al [33] (2020)	Nigeria	Review	mHealth interventions	—	App features and AI integration	Improved caregiver support and usability	Smartphone-based	Asynchronous
16	Deniz-Garcia et al [40] (2023)	UK	Scoping review	Implementation barriers	—	Cost access and policy limitations	Policy recommendations	Internet-based	Asynchronous
17	Arora et al [38] (2024)	US	Systematic review	Telemedicine platforms	—	Cognitive function QoL-AD	Reduced burden and improved outcomes	Internet-based	Synchronous
18	Scott et al [49] (2018)	Germany	Scoping review	mHealth apps	—	Adoption barriers and caregiver stress	High mobile penetration but limited rural uptake	Smartphone-based	Asynchronous
19	Hengst et al [41] (2023)	UK	Observational	Medication adherence through mHealth	120	Compliance metrics	Improved adherence and socialization	Smartphone-based	Asynchronous
20	Lim et al [50] (2018)	Spain	Observational	Remote monitoring tools	50	Cognitive tracking	Earlier interventions and better outcomes	Internet-based	Asynchronous
21	David et al [42] (2023)	UK	Survey	Specialist video consultation	—	QoL and cognitive scores	Improved outcomes in urban areas	Internet-based	Synchronous
22	Gabb et al [43] (2025)	UK	Observational monitoring study	General telehealth check-ins (wearable devices)	—	Caregiver outcomes	Reduced stress and improved satisfaction	Internet-based	Synchronous
23	Chi and Demiris [44] (2015)	UK	Review	Wearables for health tracking	—	Device accuracy	Effective monitoring and scaling potential	Wearables	Synchronous

a Not available.

b MMSE: Mini-Mental State Examination.

c HVLT-R: Hopkins Verbal Learning Test-Revised.

d mHealth: mobile health.

e QoL: quality of life.

f QoL-AD: quality of life in Alzheimer disease.

Telemedicine interventions range from remote monitoring, mHealth apps, and video consultations, reflecting differences in health care infrastructure across urban and rural Nigeria. The intervention designs were diverse, highlighting the varied nature of telemedicine adoption.

Three studies were RCTs [46,51,52]. Five studies used quantitative methods, including observational and pretest-posttest designs [6,45,47,53,54]. Six studies used qualitative methods focusing on interviews and observational data to explore feasibility, acceptability, and implementation challenges [13,34,39,48,55,56]. Nine studies used mixed methods, combining quantitative (eg, surveys) and qualitative (eg, interviews) data for comprehensive evaluation [9,11,28,33,35,41,43,49,50]

Sample sizes varied, the largest study involving 250 participants [35] and the smallest only 11 participants [37]. Three studies reported sample sizes over 100 participants [35,45,53], with larger studies using quantitative methods and smaller ones exploring qualitative insights and feasibility. Outcome measure varied, caregiver burden was assessed in 6 studies using self-reported questionnaires [6,11,32,35,39,45]. Four studies used standardized scales to measure quality of life among individuals living with dementia, such as the QoL-AD scale [6,45,46,54]. Studies in rural areas often identified internet access and digital literacy as critical secondary outcomes [6,48]. Two studies addressed behavioral and psychological outcomes, highlighting reductions in agitation and caregiver stress [34,39]. The projected telemedicine growth in Nigeria from 2018 to 2024 shows increased use of mHealth apps, video consultations, and remote monitoring tools across urban and rural areas. Studies show mHealth apps are widely used in Nigeria for telemedicine, supporting caregivers in managing care routines, monitoring individuals living with dementia’s health [33].

Common barriers include poor internet connectivity [40], high-cost platforms, devices, and mobile data for health care professionals and caregivers [38]. High mobile phone penetration supports mHealth adoption, particularly in urban areas [49]. This study highlights the challenges and benefits of telemedicine for dementia care in Nigeria, revealing gaps in infrastructure and cultural adaptation. Table 2 summarizes the studies, detailing locations, study design, sample sizes, interventions, and outcomes.

### Types of Intervention

Studies (n=23) were grouped according to the type of telemedicine intervention, including mHealth apps (n=10), video-based interventions (n=8), and remote monitoring interventions (n=5).

#### mHealth-Based Telemedicine Interventions

Ten studies assessed mHealth interventions for dementia care [11,32,36,41,45,47,52,53,55,57]. These included apps for communication, medication reminders, and stress management [45,55], as well as health-tracking tools for cognitive decline and behavior [36,47,58]. Smartphone-based systems improved usability, adherence, and caregiver support [11,32,41,52,53], though barriers such as cost, internet access, and digital literacy persist [11,52].

#### Video Consultations

Eight studies [9,13,34,35,42,46,53,58] evaluated video consultations linking individuals with dementia and their caregivers to health professionals . These interventions improved specialist care coordination, proved most effective for monitoring and assessments in urban areas, and reduced caregiver travel burden while enhancing access to specialists, although their effectiveness in rural settings was limited by internet connectivity challenges.

#### Remote Monitoring Tools

Five studies [6,34,39,41,50] investigated remote monitoring tools, including wearable sensors and home-based systems, for assessing cognitive function, daily activity, and sleep patterns. While these technologies demonstrated potential for early detection and improved care management, their adoption was hindered by costs and limited digital literacy, particularly in rural areas.

### Caregiver Support Apps

Four studies evaluated apps supporting caregivers [11,36,53,55]. These tools improved communication and reminders, reducing stress [55], enhancing well-being [53], and supporting telehealth and national strategies [11,36].

### Remote Health Tracking Apps

Three studies [36,55,58] addressed mHealth apps monitoring cognitive decline and quality of life. Yi et al [36] evaluated medication adherence and cognitive tracking, while Zou et al [57] showed reduced hospital visits, Hengst et al [41] noted improved medication adherence and socialization, while Zhu et al [47] highlighted effective symptom tracking and reduced emergency visits.

#### Video-Based Interventions

#### Teleconsultations for Specialist Care

Three studies [7,13,37] found video consultation improved care access without in-person visits. Ruggiero et al [59] reported improved quality of life and cognitive scores, especially in urban areas. Chi and Demiris [44] noted reduced stress and better patient outcomes through checkups.

#### General Remote Video Visits

Two studies examined routine video consultations for dementia care, which addressed caregiver concerns but produced mixed results [1,46]. While Mulili et al. [1] highlighted that poor connectivity limited adoption in rural areas, Page et al [46] reported reduced caregiver burden through their regular use.

#### Remote Monitoring Tools

Four studies [11,13,35,53] examined home-based and wearable sensors. Cote et al [39] evaluated wearable sensors tracking daily activities, enabling early interventions. Ojeahere et al [34] highlighted monitoring devices tracking behavioral changes and improving care.

#### Home-Based Systems

Home systems monitored daily activities, cognitive health, and behaviors, Cote et al [39] noted early detection of health declines, while Ojeahere et al [34] reported reduced caregiver workload, particularly useful in rural areas.

#### Wearable Sensors

Wearable sensors improved health tracking accuracy and interventions. Chi and Demiris [44] noted effective monitoring, while Louis et al [6] emphasized cost challenges but potential for scaling in resource-poor settings.

#### Internet Access

Rezigalla [54] and Ojeahere et al [34] discussed the challenges posed by limited internet connectivity in rural areas, which hindered the implementation of telemedicine tools. These studies suggested infrastructure improvements as a critical step to expanding telemedicine access in such regions.

#### Digital Literacy

Digital literacy was a major barrier noted by Gately et al [32] and Oyinlola et al [11]. Many caregivers and health care providers lacked the skills to use telemedicine platforms effectively, reducing their uptake. The studies recommended comprehensive training to improve digital competency and telemedicine effectiveness.

#### Challenges and Feasibility

Several studies highlighted the challenges that remain despite the benefits of remote monitoring. Louis et al [6] reported improvements in urban care management, but clinician readiness, poor internet, device costs, and legal concerns hindered rural adoption. Similar barriers were noted in Germany by Mulili et al [1], where lack of training, financial challenges, and inadequate health care infrastructure limited remote monitoring effectiveness. Travers et al [53] noted that low-resource settings faced technical literacy issues, restricting the scalability of interventions. Other interventions such as telepsychiatry and online support networks were explored [60,61]. Aderinto et al [61] studied telepsychiatry in rural Nigeria, showing potential to address mental health disparities in dementia care. Oyinlola et al [11] evaluated online support groups, noting reduced stress and caregiver burden through resource sharing and social support.

#### Telemedicine Platforms and Legal Frameworks

The scope of telemedicine platforms and regulatory frameworks affecting dementia care in low-resource settings was examined [14]. In rural areas, regulatory barriers to adoption were identified, indicating the need for supportive legal frameworks. Hamilton and Finley [56] reviewed platforms, noting that effective ones improved patient satisfaction and outcomes but were hindered by cost and accessibility issues.

#### Gaps in Research

The review identified several gaps in the current research on telemedicine for dementia care in Nigeria. First, lack of large-scale studies: larger studies are needed to assess the long-term impact of telemedicine on dementia care; most studies are small-scale [11]. Second, minimal focus on cultural adaptation: limited research addresses adapting telemedicine tools to meet the cultural needs of caregivers and health care professionals [62]. Third, absence of RCTs: few RCTs have been conducted to strengthen evidence for policy and compare telemedicine with traditional care in Nigeria [63].

## Discussion

### Summary of the Findings

This scoping review examined telemedicine interventions for dementia care in Nigeria, focusing on video consultations, mHealth, and remote monitoring tools. Video consultations effectively improved specialist access and reduced caregiver burden [11]. mHealth apps are the most used intervention due to high mobile phone penetration [63], while remote monitoring tools face challenges in rural areas due to poor internet connectivity and digital literacy gaps [3]. Key barriers include infrastructural limitations, lack of a cohesive strategy, and cultural barriers [4]. Despite challenges, telemedicine shows potential to enhance patient outcomes, reduce caregiver burden, and improve health care access [56]. The findings align with recent studies demonstrating increased adoption of digital health in low-resource settings [26,37,52]. In addition, successful implementation models, such as remote patient monitoring in dementia care, have been demonstrated in previous studies that reported the successful implementation of remote patient monitoring for dementia care [42,57].

### Detailed Discussion of Findings

Video consultations address the shortage of dementia specialists by reducing caregiver travel stress and providing timely health care access [23]. However, poor internet access and inconsistent electricity supply hinder implementation in rural areas, such as other sub-Saharan nations facing infrastructural challenges [5,53]. The extensive use of mHealth apps helps caregivers manage routines and monitor health [4]. High mobile phone penetration supports mHealth adoption, particularly in urban areas, but gaps in digital literacy limit rural use. This issue is consistent with other low- and middle-income countries facing similar telemedicine challenges [64].

Remote monitoring tools, such as wearable sensors and home-based systems, support continuous health monitoring and early detection of cognitive decline, reducing hospital visits [58]. However, affordability, digital literacy, and infrastructure challenges limit their use in Nigeria, reflecting similar issues reported globally [45,58].

Cultural and linguistic diversity also impacts telemedicine adoption. Many older adults and caregivers in rural areas are not fluent in English, the primary language of most platforms [65]. Effective adoption requires multilingual support and culturally appropriate frameworks [66]. This aligns with findings from other multicultural regions emphasizing culturally tailored interventions [65].

### Limitations

This review has limitations. The methodological quality of studies varies, with many relying on qualitative data, limiting applicability across Nigeria [18]. The review focused on published literature, overlooking relevant gray literature [52]. Few long-term studies evaluate the lasting impact of telemedicine interventions on dementia care outcomes in Nigeria, with long-term effects still unknown, despite short-term advantages [13]. Lastly, issues with internet connectivity and digital literacy are commonly noted, but strategies to address them were not comprehensively explored [41].

### Conclusions

This review highlights the urgent need for a comprehensive telemedicine policy framework integrating technological solutions within existing dementia care systems. Addressing infrastructural limitations, encouraging caregiver training, and developing culturally adapted tools are essential for broader acceptance. Caregiver-centered solutions can support a sustainable model for dementia care in Nigeria. Policymakers should focus on long-term evaluations, RCTs, and scalable models to bridge the digital divide. These strategies will enhance the quality of life for individuals with dementia and establish Nigeria as a leader in innovative health solutions for resource-limited settings.

## Supplementary material

10.2196/75168 Multimedia Appendix 1 Detailed search strategy, study selection process, and full list of included and excluded studies with reasons.

10.2196/75168 Checklist 1 Completed PRISMA-ScR 2020 checklist.

## Abbreviations

mHealth: mobile health
PRISMA-ScR: Preferred Reporting Items for Systematic Reviews and Meta-Analyses extension for Scoping Reviews
RCT: randomized controlled trial
