# Supplementary material for: Adoption of Telemedicine for Dementia Care in Nigeria: Scoping Review
Source: Interact J Med Res. 2025 Oct 27;14:e75168. doi: 10.2196/75168 (PMC12558420; doi:10.2196/75168)
Supplement: Multimedia Appendix 1 [file ijmr-v14-e75168-s001.docx]

| **Electronic databases** | **Search** | **Query** | **Records retrieved** |
| --- | --- | --- | --- |
| **PUBMED** | #1 | (“Telemedicine” OR “Telehealth” OR “Digital health” OR “Remote healthcare” OR “mHealth”) | 16,058 |
|  | #2 | (“Dementia care” OR “Cognitive impairment” OR “Alzheimer’s disease” OR “Memory loss”) | 33,681 |
|  | #3 | (“Nigeria” OR “West Africa”) | 6,760 |
|  | **#4** | **#1 AND #2 AND #3** | **130** |
| **SCOPUS** | #1 | (“Telemedicine” OR “Digital health” OR “Video consultations” OR “Remote monitoring”) | 113,590 |
|  | #2 | (“Dementia” OR “Cognitive decline” OR “Alzheimer’s disease”) | 480,240 |
|  | #3 | (“Nigeria” OR “West Africa”) | 129,477 |
|  | **#4** | **#1 AND #2 AND #3** | **150** |
| **PsycINFO** | #1 | (“Telehealth” OR “mHealth” OR “Remote healthcare”) | **9,246** |
|  | #2 | (“Cognitive health” OR “Dementia care” OR “Neurocognitive disorders”) | **3,658** |
|  | #3 | (“Nigeria” OR “West Africa”) | **12,329** |
|  | **#4** | **#1 AND #2 AND #3** | **100** |
| **CINAHL** | #1 | TITLE-ABS-KEY (“Telemedicine” OR “Remote healthcare” OR “Mobile health applications”) | 124,610 |
|  | #2 | (“Dementia care” OR “Cognitive disorders OR “Alzheimer’s disease”) | 201,908 |
|  | #3 | TITLE-ABS-KEY (“Nigeria”) | 259,662 |
|  | **#4** | **#1 AND #2 AND #3** | **120** |
| **Cochrane library** | #1 | (“Telemedicine” OR “Telehealth” OR “Remote monitoring”) | 21,855 |
|  | #2 | (“Telehealth” OR “Remote health”) AND (“Dementia”) AND (“Nigeria”) | 219,696 |
|  | #3 | (“Dementia care” OR “Cognitive disorders OR “Alzheimer’s disease”) | 114,434 |
|  | **#4** | **#1 AND #2 AND #3** | **50** |
| **Google Scholar** | **#1** | (“Telemedicine for dementia care in Nigeria”) | **50** |

A collated list of the search strategy

| **S/N** | **Databases** | **Results** |
| --- | --- | --- |
| 1 | PUBMED | 150 |
| 2 | Scopus | 130 |
| 3 | PsycINFO | 100 |
| 4 | CINAHL | 130 |
| 5 | Cochrane Library | 80 |
| 6 | Google Scholar | 50 |
| **Total** | | **640** |
